# The Impact of Chronotype on the Sleep and Training Responses of Elite Female Australian Footballers

**DOI:** 10.3390/clockssleep3040037

**Published:** 2021-10-11

**Authors:** Michele Lastella, Dean J. Miller, Manuella Quilelli, Spencer Roberts, Brad Aisbett, Dominique Condo

**Affiliations:** 1 Appleton Institute for Behavioural Science, School of Medical, Health and Applied Sciences, Central Queensland University, Adelaide, SA 5034, Australia; d.j.miller@cqu.edu.au (D.J.M.); manuquilelli@gmail.com (M.Q.); 2 School of Exercise and Nutrition Sciences, Deakin University, Geelong, VIC 3220, Australia; s.roberts@deakin.edu.au; 3 Institute for Physical Activity and Nutrition, Deakin University, Geelong, VIC 3220, Australia; brad.aisbett@deakin.edu.au; 4 Centre for Sports Research, Deakin University, Geelong, VIC 3220, Australia; dominique.condo@deakin.edu.au

**Keywords:** circadian preference, Australian football, female, sports, sleep, actigraphy

## Abstract

The primary aims of the present study were to examine the impact of chronotype on sleep/wake behaviour, perceived exertion, and training load among professional footballers. Thirty-six elite female professional football player’s (mean ± SD: age, 25 ± 4 y; weight, 68 ± 7 kg) sleep and training behaviours were examined for 10 consecutive nights during a pre-season period using a self-report online player-management system and wrist activity monitors. All athletes completed the Morningness-Eveningness Questionnaire (rMEQ) on the first day of data collection. Eleven participants were morning types, seventeen participants were intermediate types, and three participants were evening types. Separate linear mixed models were conducted to assess differences in sleep, perceived exertion, and training behaviours between chronotype groups. Morning types woke up earlier (wake time: 07:19 ± 01:16 vs. 07:53 ± 01:01, *p* = 0.04) and reported higher ratings of perceived exertion compared to intermediate types (6.7 ± 1.1 vs. 5.9 ± 1.2, *p* = 0.01). No differences were observed between chronotype groups for bedtime, time in bed, total sleep time, sleep efficiency, training duration, or training load. In circumstances where professional female football players are required to train at a time opposing their natural circadian preference (e.g., morning type training in the evening), their perceived exertion during training may be higher than that of players that are training at a time that aligns with their natural circadian preference (e.g., evening type training in the evening). It is important for practitioners to monitor individual trends in training variables (e.g., rating of perceived exertion, training load) with relation to athlete chronotype and training time. Future research should examine the relationship between chronotype, training time, and rating of perceived exertion across different training durations.

## 1. Introduction

Professional football teams are typically exposed to high-intensity training during the pre-season period to maximise adaptations prior to the competitive season. Pre-season exposes professional footballers to a range of stressors that induce a state of physiological and psychological fatigue [1]. To optimise recovery, Australian football players are often instructed to maintain consistent bed and wake-up times [2,3]. While consistent sleep/wake schedules may help improve the sleep of professional footballers, other factors such as chronotype and training load may also influence athletes’ sleep/wake behaviours. [4]. For example, previous research has shown that athletes delay their bed and wake up time on rest days compared to training days [4,5].

The circadian variation of physiological and psychological variables over the course of the day has been established over several decades [6]. Chronotype is an expression of circadian rhythmicity in an individual and can differ such that individuals demonstrate behavioural preferences to be most active in the morning or evening [6,7]. There are three different chronotypes; at one end of the scale are morning types, at the other end are evening types, while in the middle are intermediate types. Morning types are phase-advanced, being most active in the early hours of the day. Evening types tend to be most active and alert in the later part of the day, while intermediate types have a greater capacity of phase delay or advance according to their lifestyle requirements [8].

An athlete’s chronotype may influence the time of day at which their performance peaks [9,10], reflecting circadian variations in psychophysiological determinants of performance such as the perceived exertion of exercise [11], mood [12], and core body temperature [13]. For example, Anderson et al. [14] revealed that evening-type swimmers showed a reduction in performance in morning (7:00) compared to evening (19:00) time-trials. Further, post-trial levels of physiological stress were higher in both morning types and evening types when performing at times opposite to their chronotype [14]. Similar observations were found in morning-type cyclists, with higher ratings of perceived exertion when they performed high-intensity workloads in the evening compared to their morning session (Kunorozva et al., 2014). There is also evidence that the chronotype distribution of athletes within sporting teams can influence team performance [15]. Given the potential influence of chronotype on sleep propensity and athletic performance, it is important to understand the chronotype distribution within sporting teams, as well as the impact of chronotype on the sleep/wake behaviors and training tolerance of athletes. Therefore, the aims of the present study were to examine (1) the chronotype distribution in professional female Australian footballers and (2) the impact chronotype has on sleep/wake behaviour of professional footballers and their subsequent perceived exertion and training load measures.

## 2. Results

The mean chronotype score for all participants was 15.2 ± 2.9 (range, 32 to 74). Using the reduced scale of the Horne and Östberg (1976) classification system [16], 17 participants were intermediate types, 11 participants were morning types, and 3 participants were evening types. The average score obtained from the Epworth Sleepiness Scale was 6.7 ± 4.0, a score indicating no excessive daytime sleepiness. On average, participants performed between 9 and 11 training sessions during the data collection period.

**Figure 1:**
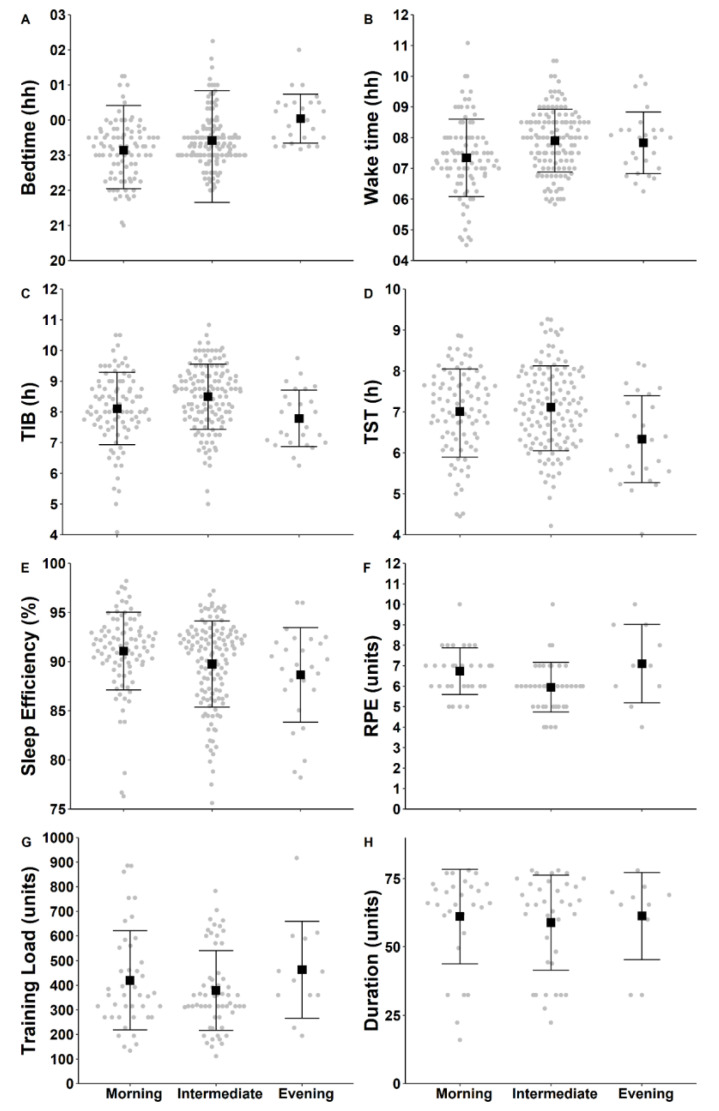
Individual data points for bedtime (**A**), wake time (**B**), time in bed (**C**; TIB), total sleep time (**D**; TST), sleep efficiency (**E**), rating of perceived exertion (**F**; RPE), training load (**G**), and training duration (**H**) as a function of chronotype. Grey dots represent individual sleep opportunities; black squares represent the group mean; error bars represent the standard deviation.

There was no main effect of chronotype on bedtime, sleep efficiency, time in bed, or total sleep time (Table 1; Figure 1). There was a main effect of chronotype on wake-up time, such that morning types woke up significantly earlier (hh:mm, 07:19 ± 01:16) compared to intermediate types (07:53 ± 01:01, *p* = 0.04). There were no differences between morning and intermediate types regarding training duration or training load (Table 1, Figure 1); however, RPE was considerably higher for morning types (6.7 ± 1.1) than intermediate types (5.9 ± 1.2, *p* = 0.01).

## 3. Discussion

The aims of the present study were to examine (1) the chronotype distribution in professional female footballers and (2) the impact chronotype has on the sleep behaviour of professional footballers and their subsequent perceived exertion and training load measures. The main findings showed that (1) few professional female Australian football players were evening types (10%), with a higher distribution of intermediate (55%) and morning types (35%); (2) wake up time was ~30 min later in intermediate types compared to morning types; and (3) morning types had higher ratings of perceived exertion during evening training compared to intermediate types.

Data examining the chronotype distribution among professional Australian football players is limited [17,18]. To the best of our knowledge, this is the first study to report the chronotype distribution of professional female football players. Similar to previous data examining their male counterparts [17], few female football players were evening types, with the majority classified as either intermediate or morning types. These data were consistent with other team sport codes such as hockey and soccer [8]. In the present study, morning types woke up significantly earlier than intermediate types. However, there were no differences between morning and intermediate types in any other sleep variable (i.e., bedtime, time in bed, sleep duration). In the context of professional sport, understanding preferences for sleep/wake timing may be a valuable tool for informing training schedules for athletes and practitioners (e.g., avoiding early morning starts for intermediate and evening types).

In the present study, morning types had a higher rating of perceived exertion during evening training session compared to intermediate types. This is consistent with previous data showing athletes’ ratings of perceived exertion were higher when training at a time opposite to their chronotype [6,11,19]. To be specific, Kunorozva et al. [11] and Mulè et al. [19] revealed morning types have higher ratings of perceived exertion when training in the afternoon compared to morning training. This evidence lends support to previous data revealing that athletes who participate in sports that match their chronotype have a higher possibility to excel [6,8]. Morning types achieved peak endurance performances in the early afternoon (i.e., midday), compared with intermediate and evening types, who peaked in the late afternoon and early evening, respectively [15]. Among morning types, increased perceived exertion during evening exercise may help explain, to some extent, sub-optimal evening performances. While this suggests that athletes may benefit from having training times tailored to their individual chronotype, more research is needed to examine the influence of chronotype on training adaptation.

In contrast to the present study’s finding that morning types had a higher perceived exertion during evening training sessions compared to intermediate types, Rae et al. [12] observed no differences in perceived exertion between morning and intermediate types at different times of the day (06:30 h vs. 18:30 h). It is plausible that Rae et al. [12] observed no differences between chronotype groups because perceived exertion was examined following a 200-metre swimming time-trial, which is considerably shorter in duration (~2.6 min). Indeed, the physiological characteristics of an Australian football training session compared to a swimming time-trial are different, with the aerobic demand on the football training session being much higher. Not surprisingly, there were no differences in the current study in training duration or training load between chronotype groups. This finding is to be expected within team sport environments because of training duration remaining consistent for the entire team. In the present study, training sessions were scheduled at specific times (i.e., ~18:00–19:30 h), and training durations were therefore similar. Indeed, in some circumstances athletes may begin training slightly earlier or stay later to perform some extra technical work, and therefore extending training duration. Despite the higher ratings of perceived exertion for morning types, no differences were observed in training load between morning and intermediate types. Given that the duration of training in this study was constant, it is unclear at which duration chronotype-related changes in RPE may start to impact internal training load. Practitioners should monitor individual trends in training variables such as RPE and training load with consideration to athlete chronotype. Future research should examine the relationship between chronotype, training time, and RPE across different training durations.

This is the first study to report (1) the chronotype distribution of female Australian football players and (2) the influence chronotype has on perceived exertion and training load of female Australian football players within a professional environment. The strengths of this study lie within the contribution to sleep and chronotype data from elite female Australian football players. Although the study had a relatively small sample size, reducing the overall power for statistical analyses, data were collected on an entire football team consisting of different types of athletes representative of a female Australian football team. Further, the use of wrist activity monitors allowed athletes to record their sleep data in their natural sleep environments, avoiding the limitations and constraints of a sleep laboratory, thus increasing the overall ecological validity of the study.

Some limitations need to be acknowledged. As a result of identifying only three evening types, analyses were restricted to comparisons between morning and intermediate types. It is important to interpret the findings related to chronotype carefully, as there was an underrepresentation of evening types. Further, without any comparative data from team-based female athletes, these data need to be interpreted with caution when generalising to other female athletic populations. It is important to acknowledge that this was an observational study, such that the direction of the effect needs to be interpreted with caution. Future research examining the impact of chronotype on the architecture of sleep in female athletes is critical for understanding the value of accommodating training programs to match athletes’ chronotype toward recovery and performance. In addition, training load was examined using the session rating of perceived exertion method [20]. The use of this method without global positioning systems may be limited to an athletes’ ability to accurately match their rate of perceived exertion with the relative level of physiologic effort [21]. Future investigations are required to assess the impact chronotype has on the physiological performance output data of female Australian football players.

### Practical Implications

There are two main implications of the present study. First, the large representation of morning and intermediate types in this study is indicative that the timing of training for female Australian football players may be suited toward the morning or middle of the day compared to evening. In addition, given wake times were typically around ~08:00 for most players, early morning training or recovery sessions should be avoided in AFLW players. While the Australian women’s football league is considered professional, it is important to note that players may not be available to train in the morning or middle of the day because of other work commitments to supplement their living. Thus, it is imperative that the football league and administrators have discussions to increase player and support staff remuneration to enable players to have full-time commitment to football, as well as more flexibility in training times. Second, training at a time that is not aligned with circadian preference (e.g., morning type training in the evening) appears to result in higher ratings of perceived exertion without changes to overall training load. Therefore, coaches and management staff working with female Australian footballers need to consider that athletes training at a non-optimal time of day for their chronotype may report higher perceived exertion. Further investigations are needed to determine if changes in perceived exertion and overall training load differ with bigger sample sizes and greater distributions across all morning, intermediate, and evening types.

## 4. Methods & Materials

### 4.1. Participants

Elite female Australian football players (female, *n* = 36; mean ± SD: age, 25.1 ± 3.8 y; weight, 86.6 ± 8.1 kg; height, 189.3 ± 7.4 cm) volunteered to participate in this study. All players were members from one team participating in the Australian Football League Women (AFLW) competition. The AFLW is the highest level of Australian rules football in Australia. Players attended an information session outlining the study requirements before informed written consent was obtained from those volunteering to participate. No participating player reported a pre-existing sleep disorder. Six participants were excluded from data analysis because of missing activity monitor or sleep diary data. The research study was approved by the Deakin University Human Research Ethics Committee (HEAG-H 182 2017; January 2019).

### 4.2. Design

An observational study design was adopted where sleep and training data were collected for 10 consecutive nights (10–19 January 2020) during a pre-season period [3]. During the monitoring period, athletes slept at their habitual place of residence, completed their normal training program, and followed their usual work/study routines. All athletes completed the Morningness-Eveningness Questionnaire (rMEQ) on the first day of data collection.

### 4.3. Sleep Assessment

Sleep behaviour was monitored using the online player-management system Smartabase (Fusion Sport, 2007–2019, Chicago, IL, USA) and a wrist activity monitor (Actical Z-series; Phillips Respironics, Bend, OR, USA). The Actical Z-series have been previously validated by Kosmadopoulos et al. [22]. Data derived from the online player-management system and wrist activity monitors were used to determine the amount and quality of sleep participants obtained. All time was scored as wake unless (1) the sleep diary indicated the player was lying down attempting to sleep and (2) the activity counts derived from the activity monitor were sufficiently low to indicate the participant was immobile. When these two conditions were met simultaneously, time was scored as sleep. This scoring process was conducted using Phillips Respironics’ Actiwatch algorithm with sensitivity set at ‘medium’ [23]. This algorithm has previously been used to quantify sleep behaviour in elite athletes [24,25]. The sleep-related dependent variables extracted from sleep diaries and activity monitors were defined as follows.

1. Bedtime (hh:mm): the self-reported clock time at which a participant went to bed to attempt to sleep.
2. Get-up Time (hh:mm): the self-reported clock time at which a participant got out of bed and stopped attempting to sleep.
3. Time in Bed (h): the amount of time spent in bed attempting to sleep between bedtime and get-up time.
4. Total Sleep Time (h): the total amount of sleep obtained during a sleep period.
5. Sleep Efficiency (%): the percentage of time in bed that was spent asleep.

### 4.4. Training Load Assessment

Training sessions included gym-based exercises and games-based training for technical and physical conditioning. All sessions included within this study commenced at 18:00 and varied in finishing times between ~19:00–19:30. Participants completed 7 to 8 training sessions out of the 10 days of data collection. Training load was monitored using the online player-management system Smartabase (Fusion Sport, 2007–2019, Chicago, IL, USA). Players recorded the duration and rating of perceived exertion (RPE) at each session. Data were then extracted by researchers, and the internal load of each session was calculated using the session RPE method [26]. Specifically, the session RPE method involves multiplying the session RPE by the duration of the session, whereby higher internal training load values are indicative of greater training demands [26].

### 4.5. Chronotype (rMEQ)

Chronotype was examined using the reduced rMEQ [16]. The rMEQ is a self-assessment questionnaire that categorises individuals based on their preference toward the performance of particular activities in the morning (e.g., At approximately what time of day do you usually feel your best?). The questionnaire consists of five multiple-choice questions and yields scores ranging from 4 to 25, with lower scores indicating participants’ preference toward evening activities, and higher scores indicating participants preference toward morning activities. Chronotype scores were determined using the rMEQ classification system (evening type, 4–11; intermediate type, 12–17; morning type, 17–25). The rMEQ has been extensively validated, showing values of Cronbach’s alpha index between 0.69 and 0.78 [16,27,28].

### 4.6. Epworth Sleepiness Scale

The Epworth Sleepiness Scale (ESS) is a scale used to assess daytime sleepiness [29]. The ESS asks the participant to rate his or her probability of falling asleep in eight different situations (e.g., sitting and reading; watching TV) on a scale ranging from 0 (no chance of dozing) to 3 (high chance of dozing). The scores for the eight questions are added to obtain a single number. A number in the range of 0–9 is considered normal, while a number in the range of 10–24 indicates excessive sleepiness, and medical advice is recommended [29]. The questionnaire has a high Cronbach’s alpha index of 0.88 [29].

### 4.7. Statistical Analyses

Data were analysed using a general linear mixed model with the R package lme4 (R Core Team, 2016, Vienna, Austria). The effect of chronotype on each dependent variable (bedtime, get-up time, time in bed, total sleep time, sleep efficiency) was examined with “condition” (morning type, intermediate type) specified as a fixed effect and “participant” entered as a random term. Evening types were excluded in the mixed-models, as only three participants were evening types [8]. The statistical significance of all fixed effects was determined using F-tests. Bonferroni corrections were made to reduce the change of obtaining a type 1 error. Statistical significance was determined with alpha set at <0.05.

## 5. Conclusions

This is the first study to examine the influence of chronotype on the sleep and training load of elite female Australian football players. Consistent with previous data from other sports, chronotype did influence ratings of perceived exertion. While training duration and subsequently training load were no different between chronotype groups, it is plausible the reduced sample size may have affected the findings. It is important for future research to increase the sample size of each chronotype group as well as using objective global positioning systems to objectively examine the influence of chronotype on physiological output, particularly at different times of the day.

## Figures and Tables

**Table 1:** Sleep and training variables as a function of chronotype.

	Chronotype			Outcomes	
Variables	Morning	Intermediate	Evening^	F (df)	*p*
Bedtime (hh:mm)	23:38 ± 01:10	23:40 ± 01:36	00:01 ± 00:42	0.01 (1,25)	0.98
Wake Time (hh:mm)	07:19 ± 01:16	07:53 ± 01:01	07:50 ± 01:00	4.72 (1,25)	0.04 *
Time in Bed (h)	8.1 ± 1.9	8.5 ± 1.1	7.8 ± 0.9	2.50 (1,25)	0.13
Total Sleep Time (h)	7.0 ± 1.1	7.1 ± 1.0	6.4 ± 1.1	0.30 (1,25)	0.58
Sleep Efficiency (%)	86.4 ± 4.0	83.5 ± 4.4	82.1 ± 4.8	1.12 (1,25)	0.30
Rating of Perceived Exertion (units)	6.7 ± 1.1	5.9 ± 1.2	7.1 ± 1.9	6.91 (1,27)	0.01 *
Training Duration (min)	61.1 ± 17.3	58.9 ± 17.4	61.3 ± 15.9	0.27 (1,26)	0.61
Internal Training load (units)	419.8 ± 201.3	378.7 ± 161.6	462.9 ± 196.5	1.02 (1,23)	0.32

Data are mean ± standard deviation. * Significant difference between conditions (*p* < 0.05).

## Data Availability

Data available on request.
